# GC/MS and LC/MS Based Serum Metabolomic Analysis of Dairy Cows With Ovarian Inactivity

**DOI:** 10.3389/fvets.2021.678388

**Published:** 2021-08-20

**Authors:** Yunlong Bai, Yuxi Song, Jiang Zhang, Shixin Fu, Ling Wu, Cheng Xia, Chuang Xu

**Affiliations:** ^1^College of Animal Science and Veterinary Medicine, Heilongjiang Bayi Agriculture University, Daqing, China; ^2^Heilongjiang Provincial Key Laboratory of Prevention and Control of Bovine Diseases, Daqing, China

**Keywords:** dairy cows, inactive ovaries, GC-TOF-MS, UHPLC-QTOF-MS, metabolism

## Abstract

Metabolic disorders may lead to the inactive ovaries of dairy cows during early lactation. However, the detailed metabolic profile of dairy cows with inactive ovaries around 55 days postpartum has not been clearly elucidated. The objective of this study was to investigate the metabolic difference in cows with inactive ovaries and estrus from the perspective of serum metabolites. According to clinical manifestations, B-ultrasound scan, rectal examination, 15 cows were assigned to the estrus group (E; follicular diameter 15–20 mm) and 15 to the inactive ovary group (IO; follicular diameter <8 mm and increased <2 mm within 5 days over two examinations). The blood was collected from the tail vein of the cow to separate serum 55–60 days postpartum, and then milked and fasted in the morning. Serum samples were analyzed using gas chromatography time-of-flight mass spectrometry technology (GC-TOF-MS) and ultra-high-pressure liquid chromatography-quadrupole-time-of-flight mass spectrometry (UHPLC-QTOF-MS). Differences in serum metabolites were identified using multivariate statistical analysis and univariate analysis. Thirty differentially abundant metabolites were identified between the two groups. In cows with inactive ovaries compared with cows in estrus, 20 serum metabolites were significantly higher (beta-cryptoxanthin (*p* = 0.0012), 9-cis-retinal (*p* = 0.0030), oxamic acid (*p* = 0.0321), etc.) while 10 metabolites were significantly lower (monostearin (*p* = 0.0001), 3-hydroxypropionic acid (*p* = 0.0005), D-talose (*p* = 0.0018), etc.). Pathway analysis indicated that the serum differential metabolites of multiparous cows in estrus obtained by the two metabolomics techniques were mainly involved in β-alanine metabolism and steroid biosynthesis metabolism, while other involved metabolic pathways were related to metabolism of glyoxylate; dicarboxylate metabolism; fructose, mannose, glutathione, glycerolipid, glycine, serine, threonine, propanoate, retinol, and pyrimidine metabolism. This indicates that the abnormalities in glucose metabolism, lipid metabolism, amino acid metabolism, and glutathione metabolism of postpartum dairy cows obstructed follicular development.

## Introduction

“Inactive ovaries” refers to two ultrasonographic examinations of the ovaries, approximately 7 days apart, revealing no substantial changes in the follicular structures, accompanied by a characteristic absence of a corpus luteum or cystic follicular structures (1). Inactive ovaries in dairy cows can cause cow anestrus, which will prolong calving intervals, increase emptying periods and breeding costs, reduce milk yield, and seriously affect the reproductive efficiency and economic benefits of cows (2).

It is well known that maternal metabolic changes can affect follicle health and oocyte development (3). There are many factors that affect the development of postpartum follicles in dairy cows, such as negative energy balance (NEB), mineral elements, vitamins, and improper management (4, 5). Early-lactation dairy cows have increased nutritional requirements, and their metabolism changes to meet the needs of postpartum mammary glands and the intake of decrease (6). If the energy intake cannot meet the body's requirements, the cow will be in an NEB. When cows are in a severe NEB, the metabolism of glucose, lipids, proteins, and other substances will be disordered. The glucose, amino acids, and lipids required for follicle growth and development will be insufficient, resulting in a lack of follicles or follicular growth, causing cows to be anestrous (7). However, the pathogenesis of inactive ovaries remains unknown.

Metabolomics generates a profile of small-molecule metabolites that are derived from cellular metabolism and can reflect metabolic network activity so that potential biological status information can be derived (8). It is worth noting that although the gene sequence is static at birth, metabolomics measurements are relatively dynamic and represent the effects of cell activity and external exposure (9). Therefore, the wealth of small-molecule metabolite data represented by individual metabolomes can generate key pathological insights.

Metabolomics has also been widely used in dairy cows over the past few years. Gerard et al. used ^1^H-nuclear magnetic resonance spectroscopy (^1^H-NMR) to perform metabonomic analysis of small and large follicular fluids of cattle and to screen four differential metabolites, including α-glucose, β-glucose, lactic acid, and tyrosine (10). Bender et al. applied gas chromatography mass spectrometry (GC/MS) metabonomics to study the metabolic differences between follicular fluid in dominant follicles of lactating cows and heifers (11).

However, metabolomics technology is still not perfect. At present, it is not yet possible to detect all compounds with one technology and different detection platforms are required. Therefore, this study combined GC-TOF-MS and UHPLC-QTOF-MS techniques to screen the differential metabolites in serum between cows with inactive ovaries and normal cows in estrus to explore the potential role of differential metabolites in follicular development, and to provide a new strategy for the prevention and treatment of postpartum inactive ovaries in dairy cows.

## Materials and Methods

### Animals

This study was administered in strict accordance with the Guide for the Care and Use of Laboratory Animals of the National Institutes of Health. All experiments on animals were carried out according to the International Guiding Principles for Biomedical Research Involving Animals. This experiment is under the supervision of the Animal Medicine Welfare Committee of Heilongjiang Bayi Agriculture University (Number of permit) SY201909005.

This experiment was carried out in a large intensive dairy farm with about 1,500 Holstein cows in Heilongjiang Province. Cows were fed a total mixed ration (TMR) in free-stall barns with continuous access to freshwater and were milked three times daily. The major ingredients of the TMR diet during early lactation were whole corn silage and oat hay.

The cows were fed a TMR diet during early lactation, which consisted of 8–9 kg of concentrate, 19 kg of silage, 3.5–4.0 kg of hay, and 350 g fat. Their nutritional level on a DM basis included 55.60% DM, 16% crude protein, 7.322 MJ·kg^−1^ net lactation production, 5.60% fat, 39.10% NDF, 20.30% ADF, 180 g of calcium, and 116 g of phosphorus. The basal diet was formulated to meet the nutrient requirements according to the Feeding Standards of Dairy Cattle in China.

Fifteen normal cows in estrus at 55–60 days postpartum (pp) were in the estrus (E) group and 15 anestrous cows at 55–60 days postpartum were in the inactive ovary (IO) group. The estrous cows had the expected signs of spontaneous estrus at 55–60 days postpartum. After rectal examination and B-ultrasound tests, the uterus did not show abnormalities. Follicular ovulation can occur on bilateral or unilateral ovaries (diameter: 15–20 mm). IO cows had no estrus performance at 55–60 days postpartum, the developmental follicle diameter was less than 8 mm, and the follicular diameter increased <2 mm within 5 days over two examinations.

After the second B-ultrasound examination, 10 ml of blood without anticoagulant was collected from the tail veins of 30 milk-producing cows 55–60 days postpartum, and then milked and fasted in the morning. Blood samples were centrifuged at 4,000g for 5 min, and 0.5 ml of serum was transferred in aliquots to a 1.5-ml EP tube and centrifuged at 12,000g for 5 min, and supernatant was transferred to a 1.5-ml EP tube and stored at −80°C.

Commercial enzyme-linked immunosorbent assay (ELISA) kits were used to measure the concentrations of the concentrations of estradiol (E_2_) and progesterone (P_4_) in 15 untreated serum samples per group. These ELISA kits were from Shanghai Hengyuan Company in China.

The background information, health status, and reproductive status of the cows were recorded through the Afimilk Cow Farm Management software.

### GC-TOF-MS Metabolite Extraction

Samples (pp 55–60d 50 μl) in 1.5-ml Eppendorf (EP) tubes were extracted using 200 μl of methanol as extraction liquid with 5 μl 2-chloro-L-phenylalanine (1 mg/ml stock in dH_2_O) added as an internal standard, followed by vortex-mixing for 30 s. Samples were incubated at −20°C for 10 min and centrifuged at 12,000 *g* for 15 min at 4°C. We then transferred the 180-μl supernatants to new 1.5-ml EP tubes to be new samples and took the 20-μl aliquot from each sample pooling to obtain a quality control (QC) sample. This was dried completely in a vacuum concentrator without heating. We then added 30 μl of methoxyamination hydrochloride (20 mg/ml in pyridine) and incubated the mixture for 30 min at 80°C. Subsequently, 40 μl of bis(trimethylsilyl)trifluoroacetamide (BSTFA) regent [1% trimethylchlorosilane (v/v)] was added to the sample aliquots and incubated for 1.5 h at 70°C. We then added 5 μl of fatty acid methyl ester (FAME) (in chloroform) to the QC sample after cooling to room temperature. All samples were analyzed using a gas chromatograph system coupled with a Pegasus HT time-of-flight mass spectrometer (GC-TOF-MS).

### GC-TOF-MS Analysis and Data Preprocessing

The processed samples were analyzed *via* GC-TOF-MS using an Agilent 7890 gas chromatography system [manufactured by Agilent Technologies, Palo Alto, CA, United States (USA)] coupled with a Pegasus HT time-of-flight mass spectrometer (manufactured by LECO Corporation, San Jose, MI, USA). We utilized a DB-5MS capillary column coated with 5% diphenyl cross-linked with 95% dimethylpolysiloxane [30 m × 250 μm inner diameter, 0.25 μm film thickness; J&W Scientific, Folsom, California (CA), USA]. A 1-μl aliquot of the analyte was injected in splitless mode. Helium was used as the carrier gas, the front inlet purge flow was 3 ml min^−1^, and the gas flow rate through the column was 1 ml min^−1^. The initial temperature was kept at 50°C for 1 min, then increased to 310°C at a rate of 20°C·min^−1^, and then kept for 6 min at 310°C. The injection, transfer line, and ion source temperatures were 280°C, 280°C, and 250°C, respectively. The energy was −70 eV in electron impact mode. The mass spectrometry data were acquired in full-scan mode with an *m/z* range of 50–500 at a rate of 12.5 spectra per second after a solvent delay of 4.70 min.

Chroma TOF 4.3X software from the LECO Corporation and the LECO-Fiehn Rtx5 database were used for raw peak extraction, filtering, and calibration of the data baseline, peak alignment, deconvolution analysis, peak identification, and integration of the peak area (12). Metabolites were identified by the mass spectrum match and the retention index match. We removed peaks when they were detected to be <50% of QC samples or where the relative standard deviation (RSD) > 30% (13).

### UHPLC-QTOF-MS Metabolite Extraction

Samples (pp 55–60d 50 μl) were thawed on ice (4°C). Next, 100 μl of the sample was placed in an EP tube and extracted with 400 μl of extraction solvent (V methanol–: V acetonitrile = 1:1), followed by vortexing for 30 s, ultrasound treatment for 10 min (with sample in ice–water), and incubation for 1 h at −20°C to precipitate proteins. The mixture was then centrifuged at 12,000*g* for 15 min at 4°C. The supernatant (425 μl) was transferred to EP tubes, and the extracts were dried in a vacuum concentrator without heating and reconstituted in 100 μl of extraction solvent [acetonitrile–water = 1:1 (v/v)]. The mixture was vortexed for 30 s, sonicated for 10 min in a 4°C water bath, and centrifuged at 12,000*g* for 15 min at 4°C. The supernatant (60 μl) was transferred to a new 2-ml LC/MS glass vial, 10 μl was taken from each sample and was pooled to create QC samples, and 60 μl of supernatant was used for UHPLC-QTOF-MS analysis.

### LC-MS/MS Analysis and Data Preprocessing

LC-MS/MS analyses were performed using a UHPLC system 1290 (manufactured by Agilent Technologies, Palo Alto, CA, USA) with a UPLC BEH Amide column (1.7 μm, 2.1^*^100 mm, Waters) coupled with a TripleTOF 6600 (Q-TOF, manufactured by AB Sciex, Framingham, MA, USA). The mobile phase consisted of 25 mM NH_4_Ac and 25 mM NH_4_OH in water (pH = 9.75) (A) and acetonitrile (B), and this process was carried out with an elution gradient, delivered at 0.5 ml·min^−1^, as follows: 0 min, 95% B; 0.5 min, 95% B; 7 min, 65% B; 8 min, 40% B; 9 min, 40% B; 9.1 min, 95% B; 12 min, 95% B,. The injection volume was 2 μl. The Triple TOF mass spectrometer was used for its ability to acquire MS/MS spectra on an information-dependent basis (IDA) during an LC/MS experiment. In this mode, the acquisition software (Analyst TF 1.7, AB Sciex) continuously evaluates the full-scan survey MS data as it collects and triggers the acquisition of MS/MS spectra depending on preselected criteria. In each cycle, 12 precursor ions whose intensity is greater than 100 were chosen for fragmentation at a collision energy (CE) of 30 V (15 MS/MS events with a product ion accumulation time of 50 ms each). ESI source conditions were set as follows: Ion Source Gas 1 = 60 Psi, Ion Source Gas 2 = 60 Psi, curtain gas = 35 Psi, source temperature = 650°C, and Ion Spray Voltage Floating (ISVF) = 5,000 V or −4,000 V in positive or negative modes, respectively.

MS raw data (.wiff) files were converted to the mzXML format using ProteoWizard and processed using the R package XCMS (version 3.2). The preprocessing results generated a data matrix that consisted of retention time (RT), mass-to-charge ratio (*m/z*) values, and peak intensity. The R package CAMERA was used for peak annotation after XCMS data processing. An in-house MS2 database was applied for metabolite identification.

### Statistical Analysis and Pathway Analysis

SPSS 19.0 software was used to perform one-way ANOVA on the basic clinical information and blood test items of selected 30 test cows. The results are expressed as “mean ± SD,” and statistical significance was defined at *p* < 0.05, with highly significant values at *p* < 0.01.

The normalized data (.txt file) were subjected to multivariate statistical analysis using R software (version 3.02) (14), which included principal component analysis (PCA) and supervised orthogonal signal correction partial least-square discriminant analysis (OSC-PLS-DA). A repeated 10-fold cross-validation and a permutation test (*n* = 200) were applied in the OSC-PLS-DA model. The quality of the model was evaluated by R^2^ and Q^2^, which indicated the total explained variation and represented the model predictability (15).

Significantly differential metabolites were screened using variable importance in projection (VIP) scores (VIP > 1) obtained from the OPLS-DA model and for *p* < 0.05.

Metabolic pathway analysis (MetPA) and hierarchical cluster analysis (HCA) were performed by MetaboAnalyst 3.0 (http://www.metaboanalyst.ca) to indicate a disturbed metabolism and construct network diagrams based on the correlations observed between differentially regulated metabolites.

## Results

As shown in Table 1, the postpartum daily milk yield of the cows in the estrus group was significantly lower than that of the cows with inactive ovaries (*p* < 0.05). Compared with cows in the estrus group, the serum E_2_ and follicle diameter concentration of cows in the IO group reduced significantly (*p* < 0.01). Age, parity, BCS, and P_4_ showed no significant differences between the two groups (*p* > 0.05). These data implied that higher milk yield and lower E_2_ level were related to the postpartum inactive ovaries during early lactation in high-producing dairy cows.

**Table 1:** Information of inactive ovaries and estrus cows in 55–60 days.

**Parameters**	**Estrus (E)**	**Inactive ovaries (IO)**
Age	2.95 ± 2.24	2.59 ± 1.60
Parity	2.09 ± 1.75	1.92 ± 1.32
BCS	3.00 ± 0.49	2.96 ± 0.42
Milk yield (kg/day)	28.96 ± 3.93	34.39 ± 7.71^*^
Follicle diameter (mm)	15.00 ± 2.27	6.00 ± 1.85^**^
E_2_ (pg/ml)	82.35 ± 5.89	46.09 ± 3.76^**^
P_4_ (ng/ml)	0.30 ± 0.08	0.28 ± 0.13

* *p < 0.05;*

** *p < 0.01. BCS, body condition score; E_2_, estradiol; P_4_, progesterone*.

### GC/MS

**Figure 1:**
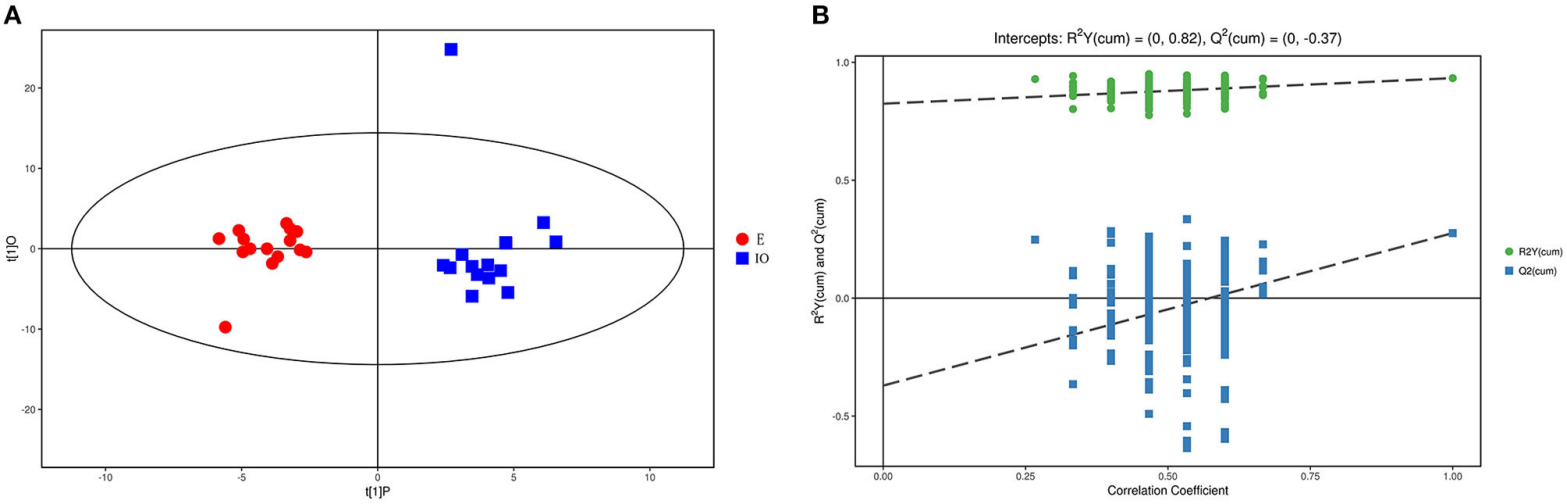
Score scatterplot of the OPLS-DA model and the permutation test for the estrus E (red) vs. inactive ovaries IO (blue) group. **(A)** A scatterplot whereby the abscissa represents the predicted score of the first principal component; the ordinate represents the orthogonal principal component score, and the scatter colors represent different experiment groups. **(B)** Result of the displacement test whereby the abscissa indicates the displacement retention; the ordinate indicates the value of R^2^Y or Q^2^, the green dot indicates the R^2^Y value, the blue square indicates the Q^2^ value, and the dotted line indicates the regression line.

As the PCA results were not significantly separated between the two groups, orthogonal partial least square discriminant analysis (OPLS-DA) was performed. The results are shown in Figure 1A. Red represents the estrus group, and blue represents the IO group. The two groups are clearly separated and are basically at the 95% confidence interval. The displacement test result is shown in Figure 1B, and R^2^Y is 0.82. As the replacement retention decreases, the proportion of the Y variable increases, and Q^2^ gradually decreases. This shows that the original OPLS-DA model is robust and fits the sample data, reflecting the real situation accurately.

Univariate analysis was used to statistically analyze the metabolites, and a total of 16 serum differential metabolites were screened (Table 2). There were nine metabolites for the IO group that were increased relative to the estrus group, which were 6-phosphoglyconic acid (*p* = 0.0368), D-fructose-2,6-diphosphate (*p* = 0.0227), D-galactonic acid (*p* = 0.0159), oxamic acid (*p* = 0.0321), alpha-tocopherol (*p* = 0.0211), 24,25-dihydrolanosterol (*p* = 0.0037), 1-methylhydantoin (*p* = 0.0485), and elaidic acid (*p* = 0.0487). There were seven metabolites that were comparatively reduced, namely, glyceric acid (*p* = 0.0131), monostearin (*p* = 0.0001), 3-hydroxypropionic acid (*p* = 0.0005), D-talose, 5-oxoproline (*p* = 0.0018), glutathione (*p* =0.0061), and thymidine (*p* = 0.0223).

**Table 2:** Differential metabolites in the serum of inactive ovaries and estrus cows.

**ID**	**Metabolites**	**RT (min)**	**VIP****^①^**	***p*-value**^②^****	**Trend**^③^****
1	Glucose 2	10.86	1.64	0.0491	↑
2	6-Phosphoglyconic acid	13.14	1.97	0.0368	↑
3	D-Fructose-2,6-bisphosphate	12.25	1.28	0.0227	↑
4	D-Galactonic acid	11.30	2.19	0.0159	↑
5	Oxamic acid	6.83	2.39	0.0321	↑
6	alpha-Tocopherol	16.60	1.15	0.0211	↑
7	24,25-Dihydrolanosterol	18.32	2.47	0.0037	↑
8	1-Methylhydantoin	7.40	1.21	0.0485	↑
9	Elaidic acid	12.33	1.06	0.0487	↑
10	Glyceric acid	7.33	1.25	0.0131	↓
11	Monostearin	14.58	3.11	0.0001	↓
12	3-Hydroxypropionic acid	5.60	2.80	0.0005	↓
13	D-Talose	10.72	2.43	0.0018	↓
14	5-Oxoproline	8.66	2.08	0.0461	↓
15	Glutathione	12.81	1.99	0.0061	↓
16	Thymidine	13.15	1.27	0.0223	↓

① *Variable importance in the projection (VIP) was obtained from OPLS-DA models with value higher than 1.0;*

② *p-value calculated from the Student's t test;*

③ *“↓” Compared to the E group, the IO group is reduced; “↑” Compared to the E group, the IO group is increased. ID, identification; RT, retain time*.

**Figure 2:**
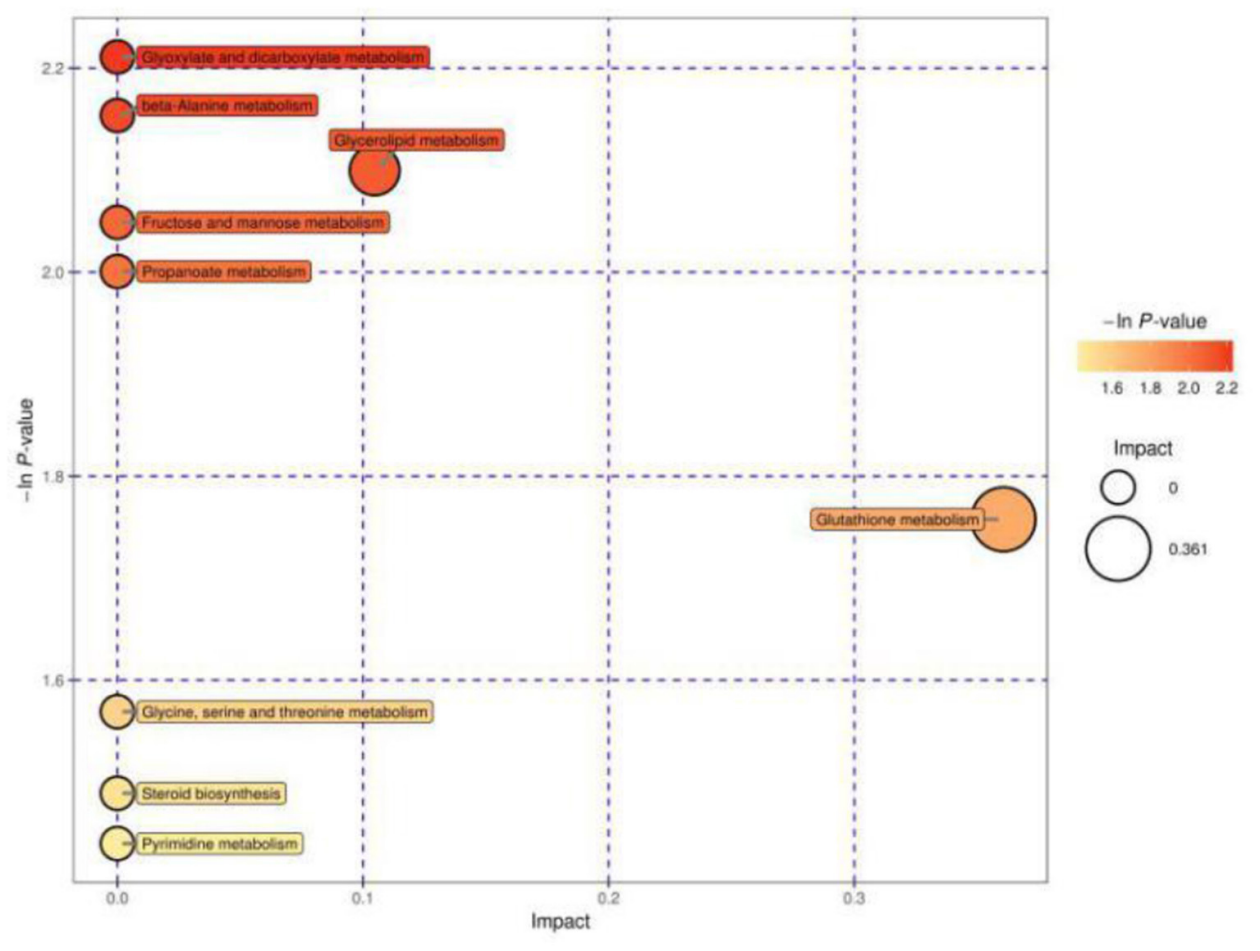
Pathway analysis for the E and IO groups. The abscissa and size of the bubble indicate the size of the influence factor of the pathway in the topological analysis. The larger the impact factor, the larger the influence. The ordinate of the bubble and the bubble color indicate the *p* value of the enrichment analysis (taking the negative natural logarithm, i.e., the –ln *P* value). The darker the color, the smaller the *p* value, and the more significant the enrichment.

Figure 2 shows the MetPA of differential metabolites between cows in estrus and cows with inactive ovaries. These pathways include glyoxylate and dicarboxylate metabolism, fructose and mannose metabolism, β-alanine metabolism, glutathione metabolism, glycerolipid metabolism, glycine, serine, and threonine metabolism, steroid biosynthesis metabolism, and propanoate metabolism. Among them, glycerolipid metabolism and glutathione metabolism are shown as darker and larger, indicating that their relative influence is greater.

### LC/MS

**Figure 3:**
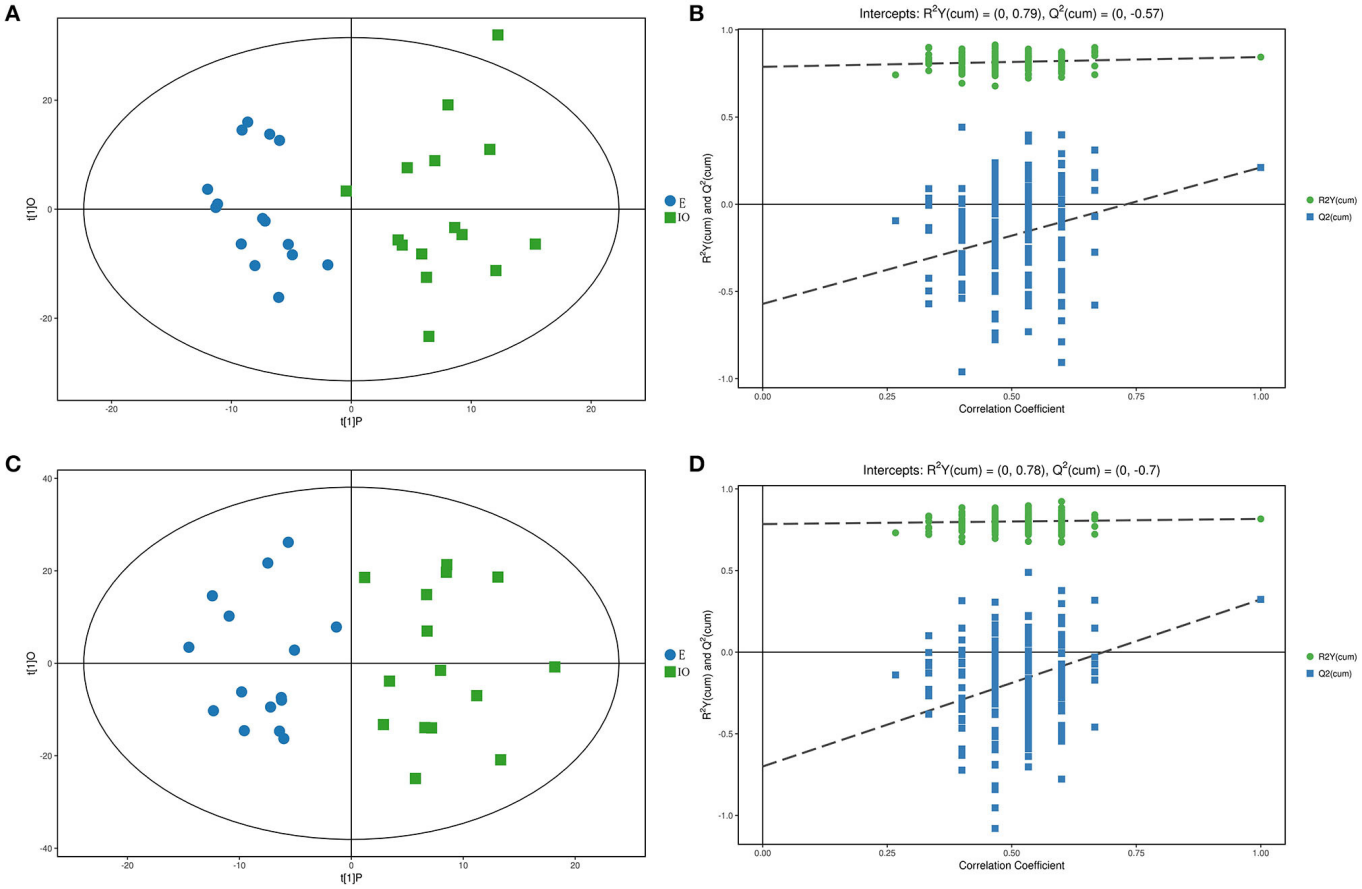
Score scatterplot and permutation test of the OPLS-DA model for the E (blue) vs. IO (green) group. **(A,B)** shows the results in ESI(+). **(C,D)** shows the results in ESI(–). **(A,C)** is a scatterplot. The abscissa represents the predicted score of the first principal component, the ordinate represents the orthogonal principal component score, and the scatter colors represent different experiments groups. **(B,D)** is the result of the displacement test. The abscissa indicates the displacement retention, the ordinate indicates the value of R^2^Y or Q^2^, the green dot indicates the R^2^Y value, the blue square indicates the Q^2^ value, and the dotted line indicates the regression line.

The PCA results were not able to significantly distinguish the two groups, so OPLS-DA was performed. The results are shown in Figures 3A,C. Blue represents the E group, and green represents the IO group. The two groups are clearly separated and are basically at the 95% confidence interval in both ESI modes. The displacement test result is shown in Figures 3B,D. R^2^Y is 0.79 and 0.78; as the replacement retention decreases, the proportion of the Y variable increases, and Q^2^ gradually decreases in both ESI modes. This shows that the original model is robust and fits the sample data, accurately reflecting the real situation.

As shown in Table 3, four differential metabolites were screened in ESI(–). Compared with the estrus group, three metabolites increased and one reduced in the IO group. Ten differential metabolites were screened in ESI(+), and compared with the estrus group, eight metabolites increased and two reduced in the IO group.

**Table 3:** Differential metabolites in the serum of inactive ovaries and estrus groups.

**ID**	**Metabolites**	**RT (min)**	**VIP**^①^****	***p*-value**^②^****	**Trend**^③^****	**ESI**^④^****
1	D-Tagatose	28.51	1.45	0.0345	↑	ESI-
2	L-Leucine-L-proline	196.76	1.49	0.0428	↑	ESI-
3	L-Anserine	399.09	1.37	0.0163	↑	ESI-
4	Cytosine	82.99	2.19	0.0496	↓	ESI-
5	Pro-Val	286.66	1.95	0.0085	↑	ESI+
6	Lys-Leu	513.86	2.15	0.0480	↑	ESI+
7	Phenylpropionylglycine	285.87	2.19	0.0056	↑	ESI+
8	Lathosterol	33.05	2.66	0.0387	↑	ESI+
9	γ-Tocopherol	32.99	1.96	0.0441	↑	ESI+
10	beta-Cryptoxanthin	32.42	2.82	0.0012	↑	ESI+
11	9-cis-Retinal	33.05	2.76	0.0030	↑	ESI+
12	Sphingomyelin (d18:1/18:0)	158.04	2.07	0.0447	↑	ESI+
13	1-Stearoyl-2-hydroxy-sn-glycero-3-phosphocholine	172.569	1.62	0.0186	↓	ESI+
14	1-Palmitoyl lysophosphatidylcholine	171.28	1.94	0.0022	↓	ESI+

① *Variable importance in the projection (VIP) was obtained from OPLS-DA models with value higher than 1.0;*

② *P-value calculated from the Student's t test;*

③ *“↓” Compared to the E group, the IO group is reduced; “↑” Compared to the E group, the IO group is increased;*

④ *“ESI-” is the negative ESI and “ESI+” is the positive ESI*.

**Figure 4:**
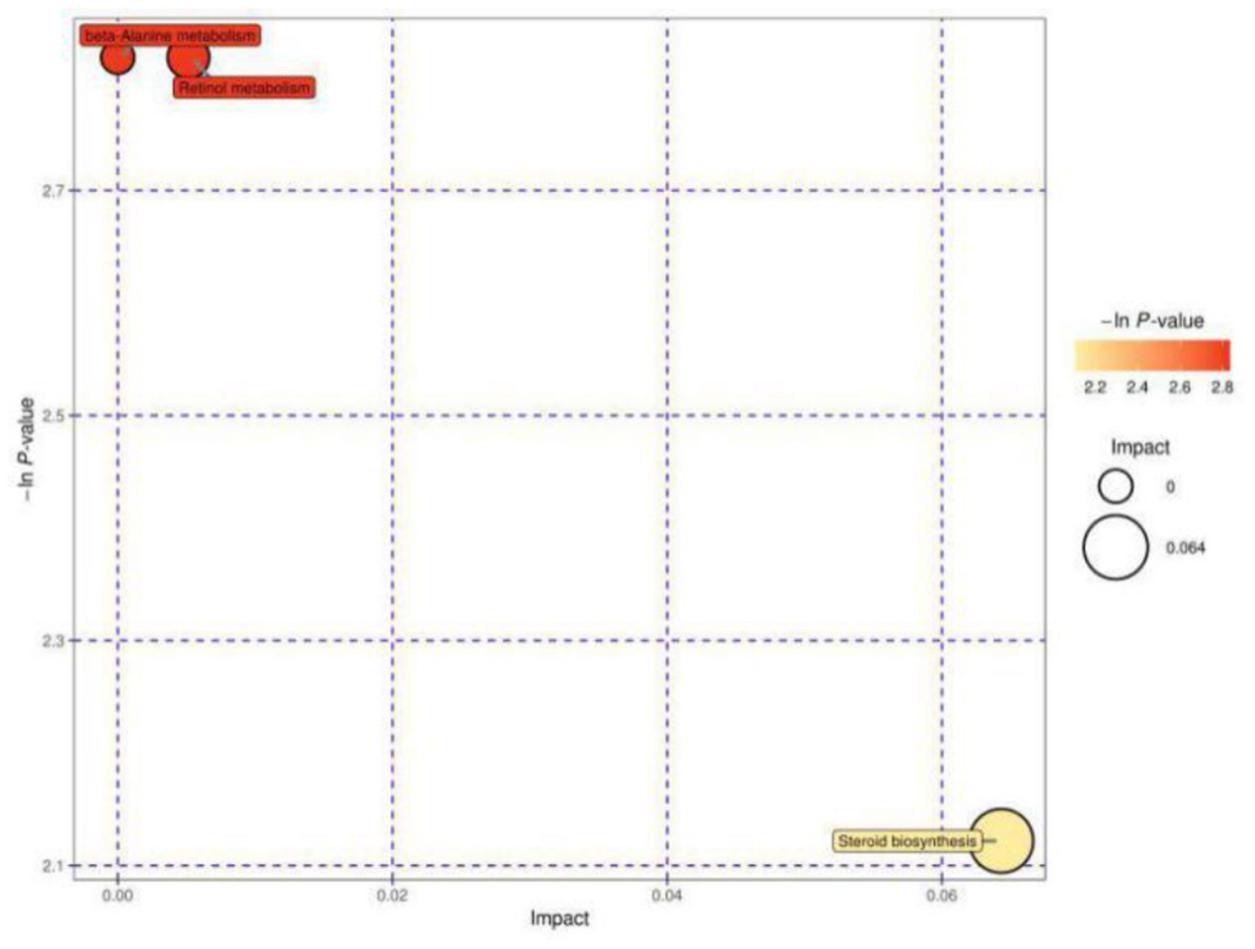
Pathway analysis for the E vs. IO group. The abscissa and size of the bubble indicate the size of the influence factor of the path in the topological analysis. The larger the impact factor, the larger the influence. The ordinate and color of the bubble indicate the *p* value of the enrichment analysis (taking the negative natural logarithm, i.e., the –ln *P* value). The darker the color, the smaller the *p* value, and the more significant the enrichment.

Figure 4 shows the MetPA of differential metabolites in estrous cows and cows with inactive ovaries, including those involved in β-alanine metabolism, retinol metabolism, and steroid biosynthesis metabolism.

### HCA Results

**Figure 5:**
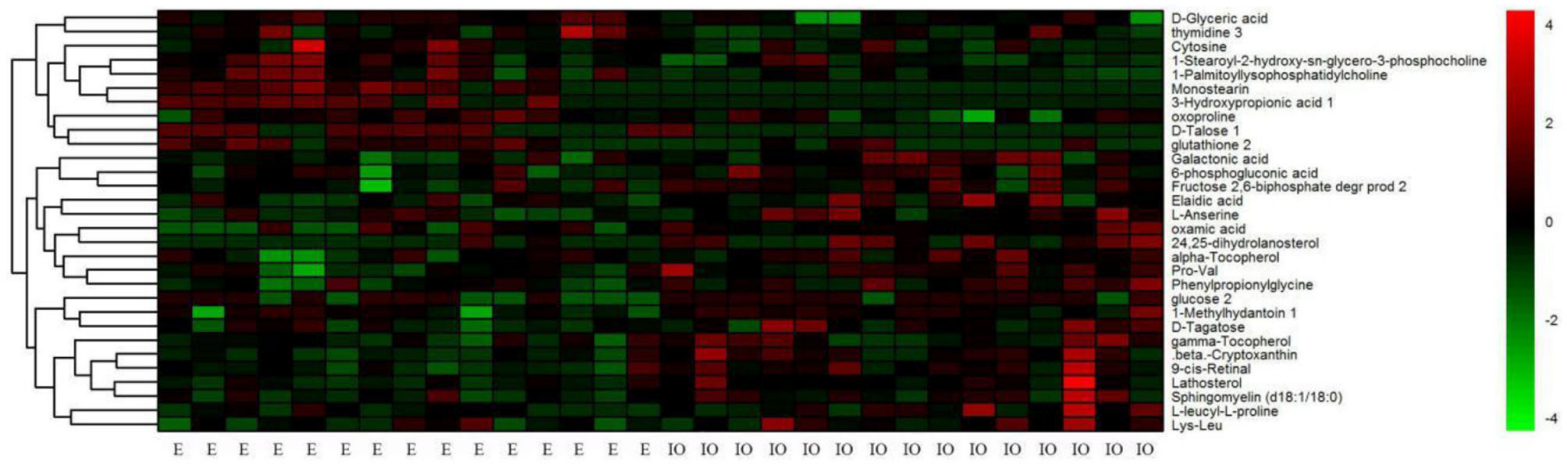
Hierarchical clustering analysis of 30 metabolites in serum from cows in estrus (the E group) and from cows with inactive ovaries (the IO group). The heat map shows the qualified metabolites using a red–black–green scheme. Red indicates that the metabolite is increased compared to the mean of the IO group, and green indicates that it is reduced. Each column represents a sample, and each row signifies an individual metabolite.

HCA based on Euclidean distances and the average linkage method was conducted to determine possible variations for the metabolite profiling of cows with inactive ovaries and cows in estrus. In common with the results from PCA, we also found that the metabolic profiles of each sample in HCA were very similar within a given group, while the metabolite profiles of the IO group were significantly distinct from those of the estrus group (Figure 5).

## Discussion

**Figure 6:**
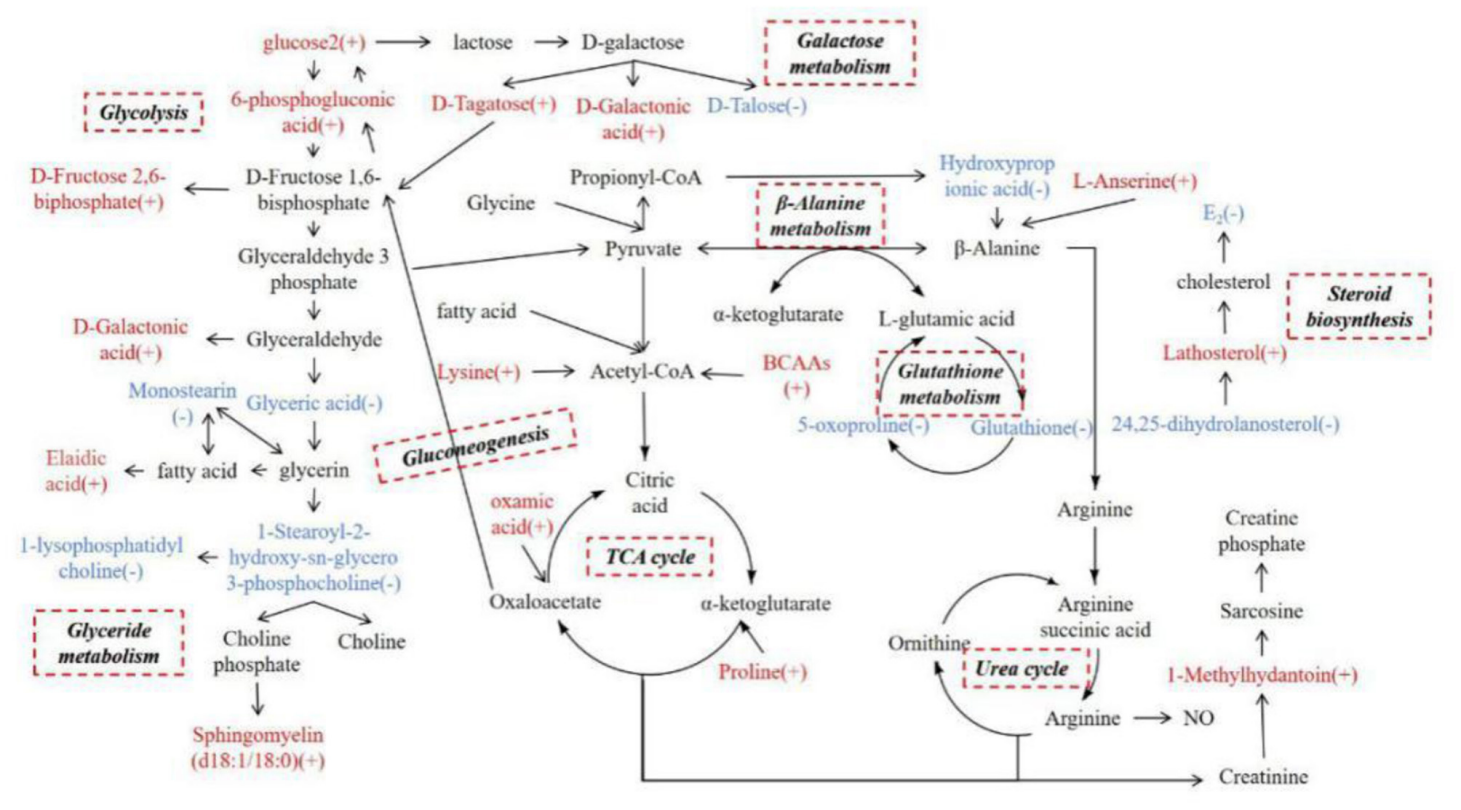
Network metabolic map for total differential metabolites, mainly for glycolysis, galactose metabolism, glyceride metabolism, the TCA cycle, the urea cycle, gluconeogenesis, β-alanine metabolism, glutathione metabolism, and steroid biosynthesis. (+) indicates higher concentrations compared with the estrous cows during inactive ovaries; (–) indicates lower concentrations.

We screened differential metabolites in the serum of cows with inactive ovaries and estrous cows by GC-TOF-MS and UHPLC-QTOF-MS. Due to the specificity, sensitivity, and metabolic libraries of the two technologies, there are some differences in the identification results. Thirty differential metabolites were identified by screening, of which 20 were increased and 10 were reduced in cows with inactive ovaries. Based on the analysis of the metabolic pathways, a network metabolic map for all differential metabolites was constructed (Figure 6). Based on the network metabolic map, the following discussion will be held.

### Glucose Metabolic Changes at 55–60 Days Postpartum in Dairy Cows With Inactive Ovaries

In the serum of cows with inactive ovaries examined in this study, glucose 2, D-fructose-2,6-bisphosphate, 6-phospogluconic acid, D-galactonic acid, and D-tagatose were found to be elevated while D-talose was reduced. Glucose can be involved in many pathways, including the glycolytic pathway, gluconeogenesis, the hexosamine biosynthesis pathway, and the pentose phosphate pathway. Regardless of the pathway, the first step is to metabolize glucose to 6-phospogluconic acid (16). 6-Phospogluconic acid is catalyzed by phosphate isomerase to form fructose-6-phosphate, and pyruvate is then generated to enter the tricarboxylic acid (TCA) cycle in the glycolytic pathway (17). The levels of glucose 2, D-fructose-2,6-bisphosphate, and 6-phospogluconic acid in serum were increased in cows with inactive ovaries. According to Table 1, the milk yield is high in cows with inactive ovaries. The increase of these metabolites may be due to the lack of energy in dairy cows, and thereby enhanced glucose metabolism supplies energy to the body and provides the energy required for lactation (18).

Glucose is preferentially supplied for lactation in cows (19). Lactose comes from blood glucose, whereby 60–85% circulating glucose is used for lactose synthesis in the ruminant mammary gland (20). Glucose is converted to galactose in the mammary gland, which then combines with glucose to produce lactose (21). Galactose is a hexose. There are two main metabolic pathways of galactose. One is to generate D-tagatose through galactitol-2-dehydrogenase, which is then converted to D-fructose-6-phosphate and enters the pentose phosphate pathway. The other is the conversion of galactose oxidase to D-galactonic acid, which in turn generates D-glycerol and enters the pentose phosphate pathway (22). In this study, D-talose, D-galactonic acid, and D-fructose-2, 6-bisphosphate were increased in the serum of cows with inactive ovaries, which suggests galactose metabolism is enhanced to present greater lactation capacity. Glucose and galactose metabolism are enhanced to meet the body's requirements for energy and lactose for lactation.

### Lipid Metabolic Changes at 55–60 Days Postpartum in Dairy Cows With Inactive Ovaries

Oleic acid, sphingomyelin, lathosterol, and 24, 25-dihydrolanostero were elevated and glyceric acid, 1-palmitoyl lysophosphatidylcholine, 1-stearoyl-2-hydroxy-sn-glycero-3-phosphocholine, and monostearin were reduced in the serum of cows with inactive ovaries. Glyceric acid, 1-palmitoyl lysophosphatidylcholine, 1-stearoyl-2-hydroxy-sn-glycero-3-phosphocholine, and monostearin in serum are involved in glycerolipid metabolism (23). Glucose enters the glycolytic pathway to produce glyceraldehyde 3-phosphate. There are then two metabolic pathways that follow. One is the generation of pyruvate in the TCA cycle, and the other is the formation of glyceric acid in glycerolipid metabolism (24). The reduction of these metabolites suggests that the metabolism from glucose to lipids is reduced in cows with inactive ovaries. When the lipid reserve decreases in these cows, lipid mobilization increases to provide energy for lactation, and elaidic acid increases. However, the relationship between the increase in sphingomyelin and inactive ovaries is unclear. In addition, lathosterol and 24, 25-dihydrolanostero can be converted into each other by the action of delta-24-sterol reductase. They are all involved in steroid biosynthesis, which are intermediate metabolites of cholesterol. They are all involved in steroid biosynthesis and play the role of many precursors of steroid hormone synthesis (25, 26). Their increase may be hindered by steroid synthesis, which leads to aggregation and inability to participate in the next metabolism. The weakened steroid biosynthesis affects the synthesis of cholesterol and sex hormones (27). It is characterized by a low E_2_ level and a small follicle diameter, which affects follicular development.

### Amino Acid Metabolic Changes at 55–60 Days Postpartum in Dairy Cows With Inactive Ovaries

In this study, L-Anserine, Lys-Leu, Pro-Val, L-Leucine-L-proline, phenylpropionylglycine, 1-methylhydantoin, and oxamic acid were increased in the serum of cows with inactive ovaries, and glutathione, 5-oxoproline, and hydroxypropionic acid were reduced. Among the increased amino acids, leucine and valine are branched-chain amino acids (BCAAs) while the others are glucogenic amino acids. They can participate in the TCA cycle by being converted into acetyl-CoA, pyruvate, and oxaloacetate. They can also enter the gluconeogenesis pathway through oxaloacetate to generate phosphoenolpyruvate to provide energy for the body (28–31). The increase in these amino acids suggests that cows with inactive ovaries mobilize protein to enhance amino acid metabolism. The increase in glucogenic amino acid especially promotes the TCA cycle to provide energy for lactation (32).

Hydroxypropionic acid and L-anserine are involved in β-alanine metabolism; the reduction of 3-hydroxypropionic and the increase of L-anserine suggest that β-alanine metabolism is weakened (33, 34).

Pyruvate produces less alanine and enters the TCA cycle for energy. Therefore, L-glutamic acid, the intermediate metabolite of pyruvate and alanine, is correspondingly reduced. L-Glutamic acid, glutathione, and 5-oxoproline participate in the glutathione cycle, resulting in a reduction in the content of 5-oxoproline and glutathione (35, 36). The antioxidant properties of GSH can protect cells from free radical damage (37). *In vitro* cysteine supplementation during embryo maturation can improve development by increasing GSH synthesis in several species. This may enhance the oocyte's antioxidant capacity and promote its development (38). Therefore, the weakened glutathione cycle may affect follicular development in dairy cows.

1-Methylhydantoin is an intermediate metabolite involved in the urea cycle of arginine, which is mainly used for the synthesis of sarcosine. Sarcosine is stored in muscle as creatine phosphate and promotes ATP synthesis (39). The increase in 1-methylhydantoin in cows with inactive ovaries may suggest that the conversion of arginine to phosphocreatine in the urea cycle is enhanced to produce energy.

### Other Metabolic Changes at 60 Days Postpartum in Dairy Cows With Inactive Ovaries

9-cis-Retinal, beta-cryptoxanthin, alpha-tocopherol, and γ-tocopherol were elevated in cows with inactive ovaries, and cytosine and thymidine were reduced. Both vitamin A and vitamin E have antioxidant effects. 9-cis-Retinal and beta-cryptoxanthin are metabolites of provitamin A, while alpha-tocopherol and γ-tocopherol are hydrolysates of vitamin E. The elevated levels of these metabolites in the serum of cows with inactive ovaries suggest beneficial antioxidative effects in the body and follicles. Tocopherol can also promote the secretion of sex hormones, increase the concentration of estrogen, and improve fertility (40). Vitamin A may affect protein synthesis through steroid hormones which, in turn, affects follicular development (41). The increase of these vitamins in IO dairy cows suggests that their utilization is blocked or that utilization is low in the early stages of follicular development, or that they are not a factor in hindered follicular development, but further confirmation is needed. In addition, cytosine and thymidine were reduced in cows with inactive ovaries, suggesting insufficient ATP in cows with inactive ovaries (42).

The amino acid metabolism disorder of dairy cows with inactive ovaries and the increase in glucogenic amino acid content observed in this study will affect glucose metabolism. Glyceride metabolism disorder results in increased lipid mobilization and limited steroid biosynthesis.

## Conclusion

This study investigated 30 differential metabolites in serum from estrous cows and cows with inactive ovaries *via* GC-TOF-MS and UHPLC-QTOF-MS. Compared with estrous cows, there were 20 increased metabolites and 10 reduced metabolites in the cows with inactive ovaries. These metabolites are mainly involved in glucose metabolism, lipid metabolism, amino acid metabolism, and other pathways. Disturbances in galactose metabolism, glyceride metabolism, steroid biosynthesis, and β-alanine metabolism were found to be the main factors, but the detailed mechanisms that affect inactive ovaries require further verification and confirmation through other experiments. According to the obtained network metabolic map regarding cows with inactive ovaries, we explored the metabolic changes and the importance of the postpartum follicular development of glucose, amino acid, lipids, and others such as glutathione, vitamin A, vitamin E, and pyrimidine in these cows. Our study provides new directions for the further exploration of the mechanisms of and prevention strategies for postpartum inactive ovaries in dairy cows.

## Data Availability Statement

The original contributions presented in the study are included in the article/supplementary material, further inquiries can be directed to the corresponding author/s.

## Ethics Statement

The animal study was reviewed and approved by Animal Medicine Welfare Committee of Heilongjiang Bayi Agriculture University.

## Author Contributions

CXi, CXu, SF, and LW designed the experiment. YB and YS conducted the experiment. YS and JZ analyzed the resulting data. YB drafted the manuscript. All authors have read and agreed to the published version of the manuscript.

## Conflict of Interest

The authors declare that the research was conducted in the absence of any commercial or financial relationships that could be construed as a potential conflict of interest.

## Publisher's Note

All claims expressed in this article are solely those of the authors and do not necessarily represent those of their affiliated organizations, or those of the publisher, the editors and the reviewers. Any product that may be evaluated in this article, or claim that may be made by its manufacturer, is not guaranteed or endorsed by the publisher.
